# Impact of HIV Infection and Zidovudine Therapy on RBC Parameters and Urine Methylmalonic Acid Levels

**DOI:** 10.1155/2016/5210963

**Published:** 2016-02-24

**Authors:** Adewumi Adediran, Vincent Osunkalu, Tamunomieibi Wakama, Sarah John-Olabode, Akinsegun Akinbami, Ebele Uche, Sulaimon Akanmu

**Affiliations:** ^1^Department of Haematology and Blood Transfusion, College of Medicine, University of Lagos, Idi-Araba, Lagos 100254, Nigeria; ^2^Department of Haematology and Blood Transfusion, National Hospital, Abuja 900241, Nigeria; ^3^Department of Haematology and Blood Transfusion, Lagos State University Teaching Hospital, Ikeja, Lagos 100271, Nigeria

## Abstract

*Background*. Anaemia is a common complication of human immunodeficiency virus (HIV) infection. The aim of this study was to investigate the impact of HIV infection and zidovudine on red blood cells (RBC) parameters and urine methylmalonic acid (UMMA) levels in patients with HIV infection.* Material and Methods*. A cross-sectional study involving 114 subjects, 94 of which are HIV-infected nonanaemic and 20 HIV negative subjects (Cg) as control. Full blood count parameters and urine methylmalonic acid (UMMA) level of each subject were determined. Associations were determined by Chi-square test and logistic regression statistics where appropriate.* Results*. Subjects on zidovudine-based ART had mean MCV (93 fL) higher than that of control group (82.9 fL) and ART-naïve (85.9 fL) subjects and the highest mean RDW. Mean UMMA level, which reflects vitamin B12 level status, was high in all HIV-infected groups but was significantly higher in ART-naïve subjects than in ART-experienced subjects.* Conclusion*. Although non-zidovudine therapy may be associated with macrocytosis (MCV > 95 fL), zidovudine therapy and ART naivety may not. Suboptimal level of vitamin B12 as measured by high UMMA though highest in ART-naïve subjects was common in all HIV-infected subjects.

## 1. Introduction

Anaemia is the most common haematological complication of HIV infection. Prevalence of anaemia in HIV infection varies from 1.3% to 95% [1–3].

Though the World Health Organization (WHO) defines anaemia as a haemoglobin concentration of <13 g/dL for men and <12 g/dL for nonpregnant women, haemoglobin cut off of <10 g/dL is often used for clinical stratification in HIV infection [4].

Anaemia in HIV infection may result from direct effect of the virus on the bone marrow through the expression of proinflammatory cytokines that suppress erythropoiesis [5]. A study in Malawi demonstrated a decreased number of both CD34+ progenitor cells and primitive erythroid progenitors in bone marrow of HIV-infected children [6]. A report has demonstrated circulating autoantibodies against endogenous erythropoietin in some HIV-infected patients due to molecular mimicry between erythropoietin and the HIV-1 p17 protein blunting the normal physiologic cytokine response to anaemia [7]. Other causes of anaemia in HIV infection include opportunistic infections, nutritional deficiencies from iron, folic acid, and vitamin B12, and toxicities from medications such as antiretroviral therapy (ART) [4, 8].

Highly active antiretroviral therapy (HAART) regimen (a combination of at least three drugs selected from the following main groups: nucleoside-analogue reverse transcriptase inhibitors (NRTIs), nonnucleoside reverse transcriptase inhibitors (NNRTIs), protease inhibitors (PIs), and entry inhibitors) decreases viral replication, restores immunologic function, and inhibits acceleration of HIV disease [9].

However, while some ART groups positively impart FBC parameters, NRTIs, most especially zidovudine, the first drug approved for the treatment of HIV infection, has been associated with anaemia [10], increase in MCV [11] and RDW [12], and other haematological disorders by a mechanism attributed to inhibition of proliferation of blood cell progenitor cells in a time- and dose-dependent fashion [9].

HIV-infected patients may also develop anaemia from nutritional deficiency such as vitamin B12 deficiency due to poor intake from reduced appetite and loss of nutrients from diarrhoea or from vomiting due to medication [13]. Measuring serum levels of vitamin B12 is problematic and estimation of UMMA has been found to be of great value in the diagnosis of vitamin B12 deficiency [14]. Except for methylmalonyl-coA mutase deficiency which can be diagnosed early in life, vitamin B12 deficiency is the only known cause of high UMMA with normal patients excreting about 0–3.4 mg/day [15].

This study was carried out to compare haemoglobin concentration, MCV, RDW, and UMMA levels in ART-naïve HIV-infected subjects, HIV-infected subjects on zidovudine-based ART, and non-zidovudine-based therapy with HIV negative subjects as control.

## 2. Materials and Methods

This cross-sectional study was carried out at the HIV Clinic of the Lagos University Teaching Hospital after approval was obtained from the Institution Research and Ethics Committee. Consented participants filled structured questionnaires which included demographic information and history of antiretroviral therapy. This cross-sectional study was carried out between March and April 2008, and subjects recruited were all those who willingly gave written consent and met the inclusion criteria during the study period. HIV negative control subjects were all those tested negative for HIV I and II by ELISA method within this same period. A total of 114 subjects were recruited, and 20 subjects were HIV negative, as controls. The remaining 94 subjects comprised ART-naive HIV-infected subjects (*n* = 29), HIV-infected subjects on zidovudine-based ART (*n* = 31), and subjects on non-zidovudine based ART (*n* = 34). The ART-experienced subjects were those who had WHO clinical stage I HIV disease and had received ART regimen for at least six months. Zidovudine-based ART consists of zidovudine, a nucleoside reverse transcriptase inhibitor (NRTI), at a dose of 300 mg twice daily and another NRTI such as lamivudine 150 mg twice daily or abacavir 300 mg twice daily in combination with a nonnucleoside reverse transcriptase inhibitor (NNRTI) such as nevirapine 200 mg twice daily or efavirenz 600 mg daily. A non-zidovudine-based ART consisted of two NRTIs and one NNRTI.

All participants in this group had baseline haemoglobin above 10 gm/dL on recruitment. HIV infection was diagnosed in blood samples using a rapid diagnostic kit followed by Western blot techniques for confirmation of HIV infection. Adherence was assessed by drug pick-up rate. Red blood cell parameters, Hb concentration, red blood cell count, MCV, MCH, MCHC, and RDW, were measured within six hours of collection by Sysmex autoanalyzer; KX-21N, Sysmex Corporation, Kobe, Japan. HIV confirmation done at registration and CD4 cell count results were within two week of this study, retrieved from the subjects' medical records. Participants with comorbid conditions such as tuberculosis and malaria were excluded from the study.

Twenty-four-hour urine sample was also collected from each participant into two-litre containers and stored at −20°C until being ready for UMMA assay. UMMA assay was done using cation exchange High Performance Liquid Chromatography (HPLC) (Agilent) series 1100 (Japan) with ultraviolet detector (wavelength 230 nm); ODS Hypersil column (reverse phase C18 and length 250 mm by 4.6 mm) with particle size of 5 microns and an ambient temperature. The HPLC's mobile phase was acetonitrile/8 mmol H_2_SO_4_ (ratio 20/80%); flow rate was 0.7/mL with a manual injector (20 *μ*L loop) and isocratic elution.

### 2.1. Data Analysis

Data entry and analysis were performed using the SPSS version 16 (SPSS Inc., Chicago). The descriptive data were given in percentages and descriptive data presented in simple tables of percentages and association tested by Chi-squared test and simple logistic regression for dichotomous responses. The differences were considered to be statistically significant when the *p* value (ANOVA) was less than 0.05.

## 3. Results

Data obtained from 114 subjects comprising 40 males (35.0%) and 74 females (65.0%) were evaluated. The subjects were divided into four groups—20 HIV negative subjects as control, subjects who were treatment-naïve (*n* = 29), subjects on zidovudine-based HAART (*n* = 31), and subjects on non-zidovudine-based HAART (*n* = 34).

The age and sex distribution of the subjects is shown in Table 1. Female subjects (*n* = 74) almost double male subjects (*n* = 40) and the age group with the highest frequency was 30–39 years (41.2%). This was followed by 40–49-year age group (26.3%). Female subjects were not significantly older than male subjects (mean age: F = 41.14 ± 10.69; M = 38.95 ± 9.74). Comparison of mean values of Hb, MCV, RDW, UMMA, BMI, and CD4 parameters among various groups is presented in Table 2. Using Hb < 10 g/dL as cut-off, none of the three groups of HIV-infected subjects met the criteria that define anaemia. However, the mean Hb concentration of subjects on zidovudine-based ART (10.1 g/dL) was close to the cut-off value and was significantly lower than the mean Hb concentrations of other groups (naïve, 11.4 g/dL; non-zidovudine-based, 11.7 g/dL); *p* = 0.008.

The lowest mean MCV was found in the control group (82.9 fL). The mean MCV of treatment-naïve subjects (85.9 fL) was significantly lower than treatment experienced subjects (zidovudine-based, 93.0 fL; non-zidovudine-based, 97.4 fL); *p* = 0.001.

The mean RDW-SD was significantly higher in the treatment experienced subjects (zidovudine-based, 58.5 fL; non-zidovudine-based, 55.8 fL) than both treatment-naïve (50.6 fL) and the control group (43.5 fL) with zidovudine-based group having the highest RDW; *p* = 0.028.

Table 2 also shows that the mean UMMA in HIV negative subjects (2.85 mg/24 hrs) was the lowest among the four groups. Mean UMMA was highest in treatment-naïve subjects (40.0 mg/24 hrs) and it was significantly higher than in treatment experienced (zidovudine-based, 10.2 mg/24 hrs; non-zidovudine-based, 7.7 mg/24 hrs) subjects; *p* = 0.001. Of the treatment experienced subjects, those on zidovudine-based ART had a marginal higher UMMA (10.2 mg/24 hrs) than non-zidovudine-based ART combination (7.7 mg/24 hrs).

The mean BMI, red blood cell count, MCH, and MCHC were not significantly different among subject groups.

Logistic regression analysis of factors influencing elevated UMMA among subjects is shown in Table 3. Untreated HIV infection was closely associated with elevated UMMA (OR = 6.125; *p* = 0.001), while treatment with either zidovudine- or non-zidovudine-based ART was associated with significantly lower UMMA (OR = 0.403; *p* = 0.002).

Table 4 shows the association between UMMA, MCV, and treatment categories. The 24-hour UMMA level at 95th percentile distribution for control subjects was 15.76 mg/24 hrs. The proportion of treatment-naïve subjects with UMMA above 15.76 mg/24 hrs were significantly higher than those with UMMA below 15.76 mg/24 hrs (69% versus 31%). However HIV positive subjects on treatment showed a significant reversal in this trend with 82% and 84% of HIV positive subjects on zidovudine-based and non-zidovudine-based treatment options, respectively, having UMMA levels below the 95th percentile value of HIV negative controls. The MCV of subjects associates significantly with treatment options as 59% of HIV positive subjects on non-zidovudine-based therapy tended towards macrocytosis (MCV > 95 fL).

## 4. Discussion

In this study, female subjects almost doubled the male subjects. This ratio is similar to a report on gender infection ratio from South Africa in which the infection rate in the 15–24-year age group is estimated to be 20 women for every 10 men. A report by Akinbami et al. [16], however, gave a male : female ratio 1 : 1.6 in Lagos, Nigeria, while another report from Makurdi in Northern Nigeria gave a male to female ratio of 1 to 1.3 [17]. This gender difference underscores the role of gender inequalities on women's risk and vulnerability to HIV infection which may be due to differences in biology and social behaviour between both sexes. According to World Health Organization (WHO) [18], women have a larger surface area of mucous membrane that is exposed during sexual intercourse than men and, unlike men, they are also exposed to larger quantity of infectious fluids (semen). In Africa, a woman is unable to negotiate safe sex and though social norm does not allow her to practice extramarital and premarital sex like man, “remaining faithful” is no protection against HIV infection [18]. Another reason for the higher number of female participants than males seen in this study might be because women are more likely to seek treatment. This is because the men are more likely to be in “denial” with the attendant fear of stigmatization leading to poor clinic attendance than the females.

Zidovudine therapy is associated with low mean Hb concentration (10.1 g/dL) close to cut-off value (<10.0 gm/dL) in our study. Though at low normal level, it agrees with reports that zidovudine is myelosuppressive [10, 19]. Perhaps more time is needed to establish the long-term effects of these drugs on RBC parameters.

Mean MCV of ART-experienced subjects was found to be higher than ART-naïve and HIV negative subjects. However, contrary to our expectation based on available reports, it is only in non-zidovudine-based ART that the MCV exceeds the cut-off that defines macrocytosis using MCV > 95 fL our local laboratory reference range. In their study using MCV > 100 fL to define macrocytosis, Romanelli et al. found significant macrocytosis with the use of zidovudine [20]. Though our mean MCV did not reach a value that defines macrocytosis, which may be due to small sample size used, this study established that use of zidovudine is associated with increased mean MCV. It should be noted, however, that though increased MCV was also noticed in non-zidovudine-based group, there was no anaemia in this group. This finding has also been reported in [21]. In fact, some authors have suggested the use of increasing MCV [20] and RDW [12] as simple and cheap-to-measure markers of early adherence to zidovudine therapy [12]. In addition, they also suggested that zidovudine therapy should be included in the differential diagnosis of an elevated RDW [12]. Our finding on zidovudine and elevated RDW is in agreement with this suggestion. However, contrary to available reports, anisocytosis as defined by RDW-SD ≥ 50 fL [22] was found in both zidovudine-based (58.5 fL) and non-zidovudine-based (55.8 fL) ART in our study. We did not find a similar report to corroborate this finding.

Mean UMMA level though raised in all HIV-infected groups in this study, it is found to be significantly higher in treatment-naïve than in treatment experienced subjects. This might be an indication that Vitamin B12 deficiency is commonly associated with HIV infection and may improve with antiretroviral therapy. This is not surprising because it has been widely reported that vitamin B12 deficiency is common in HIV infection [23, 24]. Higher level of UMMA in treatment-naïve subjects than treatment experienced ones found in this study is also in line with a report by Semeere et al. [25] who found a high prevalence of vitamin B12 depletion among ambulatory HIV-infected, ART-naïve adults attending their clinic. Suboptimal vitamin B12 level that still exists among the treatment experienced subjects agrees with the report of Owiredu et al. in that though HAART may improve serum B12 levels [26], vitamin B12 supplement is required as an adjunct therapy to HAART in the management of HIV infection [26].

## 5. Conclusion

Suboptimal B12 deficiency (measured by UMMA level) is commonly found in HIV infection. Though zidovudine therapy is associated with low mean Hb concentration and increased RDW, it is not associated with macrocytosis. However, nonzidovudine therapy may be associated with macrocytosis. More time is needed as a follow-up of these subjects to fully establish these findings.

## Figures and Tables

**Table 1:** Age group and sex of subjects.

Age groups (years)	Male	Female	Total
	*n* (%)	*n* (%)	
≤29	7 (44)	9 (56)	16
30–39	14 (30)	33 (70)	47
40–49	14 (47)	16 (53)	30
50–60	5 (24)	16 (76)	21
Total	40 (35)	74 (65)	114
Mean (±SD)	38.95 ± 9.74	41.14 ± 10.69	40.24 ± 10.42
*p* value			0.257

*χ* ^2^ = 4.05; *p* value = 0.257.

**Table 2:** Comparing mean values of Hb, MCV, RDW, UMMA, BMI, and CD4 parameters among study groups by ANOVA.

Parameters	Control	Naïve	Zt	NZt	*p* value
	*n* = 20	*n* = 29	*n* = 31	*n* = 34	
Hb (g/dL)	12.80	11.40	10.10	11.70	0.008
MCV (fL)	82.90	85.90	93.00	97.40	0.001
RDW-SD (fL)	43.50	50.60	58.50	55.80	0.028
UMMA (mg/24 hrs)	2.85	40.00	10.20	7.70	0.001
BMI (kg/m^2^)	25.4	24.68	22.92	23.03	0.377
CD4 (cells/*µ*L)	306	304	261	310	0.613

UMMA, urine methylmalonic acid; MCV, mean cell volume; RDW, red cell distribution width; Hb, haemoglobin concentration; BMI, body mass index; CD4, cluster of differentiation 4; naïve, HIV positive treatment naïve subjects; Zt, zidovudine based treatment group; NZt, non-zidovudine-based treatment group. *p* value (significant level for ANOVA statistics). Post hoc statistics: show significant mean difference in ZT versus NZt group for Hb, MCV, and RDW-SD (*p* < 0.05).

**Table 3:** Logistic regression analysis of factors influencing elevated UMMA among study subjects compared to HIV negative subjects.

Variables	Odd ratio	*p* value	95% CI
HIV-infected treatment-naïve	6.125	0.001	2.029, 18.493
HIV-infected on ART	0.403	0.002	0.427, 0.715

**Table 4:** Association between UMMA, MCV, and treatment categories.

	Treatment groups		
	Nt (%)	Zt (%)	Nzt (%)
UMMA (mg/24 hrs)			
≤15.76	8 (31%)	22 (82%)	27 (84%)
>15.76	18 (69%)	5 (18)	5 (16%)
Total	26	27	32
*χ* ^2^ (*p* value)			1.842 (0.000)
MCV (fL)			
≤95	24 (92)	15 (56)	15 (41)
>95	2 (8)	12 (46)	15 (59)
Total	26	27	32
*χ* ^2^ (*p* value)			1.842 (0.000)

Treatment-naïve (Nt); Zidovudine based treatment group (Zt); non-zidovudine-based treatment group (NZt); urine methyl malonic acid (UMMA); mean cell volume (MCV); Chi-square value (*χ* ^2^).
